# Effects of the surface characteristics of nanoporous titanium oxide films on Ti-24Nb-4Zr-8Sn alloy on the initial adhesion of osteoblast-like MG-63 cells

**DOI:** 10.3892/etm.2013.1104

**Published:** 2013-05-08

**Authors:** YUQUAN HAO, SHUJUN LI, XUESONG HAN, YULIN HAO, HONGJUN AI

**Affiliations:** 1 Department of Prosthodontics, School of Stomatology, China Medical University, Shenyang, Liaoning 110002;; 2 Shenyang National Laboratory for Materials Science, Institute of Metal Research, Chinese Academy of Sciences, Shenyang, Liaoning 110016;; 3 Department of Stomatology, Liaoning Provincial Corps Hospital, Chinese People’s Armed Police Forces, Shenyang, Liaoning 110034, P.R. China

**Keywords:** titanium alloy, nanoporous surface, osteoblasts, early adhesion

## Abstract

The aim of the present study was to investigate the effects of the surface characteristics of nanoporous titanium oxide films, formed by anodization on Ti-24Nb-4Zr-8Sn (Ti2448) alloy, on the early adhesion of osteoblast-like MG-63 cells. Nanoporous titanium oxide films with two different pore sizes (30 and 90 nm) were formed by anodization in NH_4_F solution on Ti2448 alloy. The surface roughness of the nanoporous titanium oxide films was determined using a Surftest Formtracer and field emission scanning electron microscopy (FESEM). Cell viability was evaluated at different time points using the 3-(4,5-dimethylthiazol-2-yl)-2,5-diphenyltetrazolium bromide (MTT) assay. To investigate the regulatory mechanisms involved in the focal adhesion of osteoblasts to Ti2448 alloy, we quantified the expression levels of integrin β1 and paxillin mRNAs on the nanoporous titanium oxide films during early osteoblast adhesion using real-time RT-PCR. Samples with a 30-nm nanoporous film exhibited a greater number of overlapping microporous structures with microprojections compared with the 90-nm nanoporous film samples. The MTT assay indicated that cell viability on the 30-nm nanoporous surface following 24 and 48 h of cell culture was higher than those observed on the unanodized control and 90-nm nanoporous surfaces. Integrin β1 mRNA expression levels on the 30-nm nanoporous surface following cell culture for 48 h were also significantly higher compared with those on the unanodized control and 90-nm nanoporous surfaces. The results demonstrated that a 30-nm nanoporous titanium oxide film on Ti2448 alloy may provide the optimum bioactive implant surface for the initial adhesion of osteoblasts.

## Introduction

The future use of titanium alloys as implant materials is predicted to increase due to their good biocompatibility, mechanical properties and chemical stability (1). Titanium alloys are also lightweight, possess high toughness and tensile ductility, a high corrosion resistance and the ability to withstand extreme temperatures (2). Titanium alloys are roughly classified into four categories depending on their purity and crystal structures: commercially pure titanium (CP-Ti); α alloy (body-centered cubic structure, BCC); β alloy (hexagonal close-packed structure, HCP) and α + β dual-phase alloy (3). Compared with α and α + β types, β-type titanium alloy offers a more advantageous material for dental implants due to its improved mechanical properties, higher corrosion resistance and a lower modulus of elasticity (4). Ti-6Al-4V, an α + β type titanium alloy, is the most widely used titanium alloy for medical implants, including orthopedic, dental and cardiovascular implants (5). However, the elastic modulus of Ti-6Al-4V alloy is 4–10 times that of human bone, and an elastic modulus mismatch between bone and implant material has often been cited as a cause of implant failure (6). Furthermore, the elements Al and V of Ti-6Al-4V alloy are capable of being released into tissue cells by passive film dissolution, which may induce Alzheimer’s disease, neuropathy, osteomalacia and allergic reactions. Therefore, novel titanium alloys containing non-toxic and non-allergic elements, including Nb, Ta, Zr, Mo and Sn, have been designed and constructed for biomedical applications (7). The novel β-type titanium alloy Ti-24Nb-4Zr-8Sn (Ti2448) has attracted considerable attention due to its high strength, high fatigue resistance and a low elastic modulus (8). An *in vivo* study of Ti2448 in New Zealand white rabbits demonstrated that the low elastic modulus of Ti2448 leads to significant improvements in new bone formation following a tibial shaft fracture compared with a Ti-6Al-4V alloy (9). These results suggest that intramedullary nails constructed of low modulus Ti2448 alloy improve new bone formation in the marrow cavity during the initial stages of bone healing.

Since titanium is biologically inert and does not bond directly nor immediately to the bone following implantation, electrochemical anodic oxidation has been applied to improve a number of the surface characteristics of titanium, including corrosion resistance, cell proliferation, cell adhesion and cell viability (10). Anodic oxidation is generally considered as a useful method for modifying the surface structure of titanium in several ways, including corrosion protection, aspect improvement and bonding of polymers (11). The anodic oxidation technique is capable of forming a nanoporous titanium oxide film of controllable pore size, good uniformity and conformability over large areas at low cost (12). Titanium oxide films with nanoporous structures are desirable for these applications due to their large surface areas and high reactivity (13). It has also been reported that the interactions between osteoblasts and titanium oxide films involve chemical reactions (14). Greater surface roughness and surface energy, more surface hydroxyl groups and the presence of fibronectin or vitronectin are important elements for the adhesion, spreading and proliferation of osteoblasts on titanium surfaces (14,15). However, the majority of studies have focused on osteoblast adhesion using a sol-gel derived hydroxyapatite coating on Ti-6Al-4V alloy (16–18). Few studies have been published with regard to nanoporous titanium oxide films on Ti2448 alloy formed by anodic oxidation with a typical pore size <100 nm. Therefore, we hypothesize that nanostructured oxide films may enhance osteoblast cell adhesion on Ti2448 alloys.

In the present study, the effects of the surface characteristics of nanoporous titanium oxide films on the initial adhesion of osteoblast-like MG-63 cells was investigated. Nanoporous titanium oxide films with two different pore sizes (30 and 90 nm) were formed by anodization in NH_4_F solution on Ti2448 alloy. Titanium surface roughness was examined using a Surftest Formtracer and field emission scanning electron microscopy (FESEM). Cells were evaluated for cell viability at different time points using the 3-(4,5-dimethylthiazol-2-yl)-2,5-diphenyltetrazolium bromide (MTT) assay. To investigate the regulatory mechanisms involved in the focal adhesion of osteoblasts to Ti2448 alloys, the expression levels of integrin β1 and paxillin mRNAs in response to the surface structure of nanoporous titanium oxide films during the initial osteoblast adhesion were quantified using real-time RT-PCR. The present study may serve as a foundation for the development and clinical application of Ti2448 alloy as a novel implant material.

## Materials and methods

### Preparation of nanoporous titanium oxide films on Ti2448 alloys

Ti2448 alloys were purchased from Shenyang National Laboratory for Materials Science, Institute of Metal Research, Chinese Academy of Sciences (Shenyang, China). Disks of 10-mm diameter [used for scanning electron microscopy (SEM) analysis and in the MTT assay] and 30-mm diameter (used for real-time RT-PCR analysis) were cut from Ti2448 alloys using a diamond cut-off wheel (Struers, Glasgow, UK). Prior to electrochemical anodization, the sheets were ground using 2400-grit emery paper (Softflex, Bad Säckingen, Germany) and polished with diamond paste (6 *μ*m). The samples were then degreased by sonication for 10 min in acetone, isopropanol and methanol; following this, the samples were rinsed with deionized water, dried in a nitrogen stream and stored covered under UV light (19).

Anodic oxidation treatment of Ti2448 alloys was performed at room temperature in a neutral electrolyte with 1 mol/l (NH_4_)_2_SO_4_ and 0.15 mol/l NH_4_F (pH 6.7) prepared from analytical grade chemicals and deionized water. A direct current power supply was used to keep the potential at a constant value. On several occasions, the potential was swept from the open circuit potential to the desired final potential with a sweep rate of 0.5 V/sec. A two-electrode system, with stainless steel as a cathode and sample as an anode, was used to form the nanoporous titanium oxide films under non-stirred conditions. All samples were cleaned using deionized water following the anodization process.

Anodic oxidation was performed at potentials of 10 and 25 V, and TiO_2_ nanotubes with diameters of 30 and 90 nm were formed in turn. The anodized samples were named as the 30- and 90-nm groups, respectively. Samples without anodic oxidation treatment were used as the control. All samples were cleaned ultrasonically in acetone, alcohol and deionized water (each for 10 min), and sterilized by cobalt-60 irradiation prior to cell culture (20,21).

### Surface morphology

The anodized specimens were uniformly coated with a layer of gold for electric conductivity and the morphology of the nanoporous titanium oxide films was observed using FESEM (LEO model Supra 35, Oberkochen, Germany) (22). Roughness was measured using a Surftest Formtracer (Surftest SV-402; Mitutoyo Instruments, Tokyo, Japan) on three samples of each group. For each sample, five profiles were recorded (23). ANOVA was performed to test the homogeneity of the anodization process of each sample and the reproducibility of this process between samples of each group. ANOVA was then applied to the usual roughness parameters (R_a_, arithmetic mean deviation of the roughness profile; R_z_, mean peak-to-valley height; R_y_, maximum height of the roughness).

### Cell culture

Osteoblast-like MG-63 cells (CRL-1427; American Type Culture Collection, Manassas, VA, USA) were cultured in Dulbecco’s modified Eagle’s medium (DMEM) (Invitrogen Life Technologies, Carlsbad, CA, USA), supplemented with 2 mM L-glutamine (Invitrogen Life Technologies), 10% fetal calf serum (FCS; Eurobio, Paris, France) and 50 *μ*g/ml gentamicin sulfate (Sigma, St. Louis, MO, USA). Cultures were maintained at 37°C in a humidified incubator in the presence of 5% CO_2_ and subcultures were performed using a 0.01% trypsin solution in phosphate-buffered saline (PBS) at pH 7.4. The cultures were observed daily under an inverted microscope (magnification, ×400). Media were changed every other day. The cells were routinely passaged until they reached 80% confluency (14).

The titanium disks were placed in a 24-well plate with a cell density of 5,000/cm^2^ (for SEM analysis and in the MTT assay) or a 6-well plate with a cell density of 50,000/cm^2^ (for real-time RT-PCR analysis). The cells were cultured for different incubation periods according to the study design.

### Cell morphology

Following 48 h incubation, the cultured disks were rinsed three times with PBS and fixed with 2.5% glutaraldehyde diluted in 0.1 M PBS for 2 h at 4°C, postfixed in 1% osmium tetroxide, dehydrated in graded ethanol series, treated with hexamethyldisilazane (Sigma) and then subjected to critical point drying. A thin layer of palladium-gold was sputter coated onto the samples prior to examination by FESEM (24).

### MTT assay

MTT is transformed by mitochondrial dehydrogenases into formazan, enabling mitochondrial activity and cell viability to be assessed. Following incubation, the samples were washed with PBS and transferred to a new 24-well plate. Then, 300 *μ*l culture medium and 300 *μ*l MTT reagent (5 mg/ml in PBS; Sigma) were added to each well. Following 4 h incubation in a 5% CO_2_ incubator at 37°C, the medium was replaced with 500 *μ*l dimethyl sulfoxide to dissolve formazan. The plate was shaken for 10 min and then the solution in each well was transferred to a 96-well ELISA plate. The optical density (OD) value of the dissolved solute was measured using an ELISA reader (Tecan, Grödig, Austria) at 570 nm (n=9 for each group). The common OD value of the blank group was subtracted from the OD value in each group at each time point (n=9). The blank group was treated with the same methods and incubated for the same time as those in the above three groups (25).

### Real-time RT-PCR assay

Following 24 and 48 h incubation, the samples were washed with PBS and transferred to a new 6-well plate. Total RNA was extracted from the cultured cells using the TRIzol^™^ reagent (Invitrogen Life Technologies) according to the manufacturer’s instructions. First strand cDNA was synthesized from 10 *μ*g total RNA using the PrimeScript^™^ first strand cDNA synthesis kit (Takara Bio, Inc., Osaka, Japan). PCR primers were designed using Primer Express 2.0 software (Applied Biosystems, Foster City, CA, USA). The primer sequences and products of selected genes for real-time PCR are summarized in Table I. Real-time PCR was used to quantify the gene expression levels of integrin β1 and paxillin when the cells had been cultured for 24 and 48 h, and the standard PCR cycle threshold (Ct) method was performed using the Roche Molecular Biochemicals LightCycler instrument (Roche Diagnostics GmbH, Mannheim, Germany).

### Statistical analysis

Data are presented as mean ± SD, median with interquartile ranges (IQR) or frequencies. A χ^2^ test was used to compare frequencies. One-way ANOVA and the Student’s t-test were used for normally distributed variables, and the Mann-Whitney U test was used for non-normal distributed variables. Comparisons between two groups for nominal variables were performed by Fisher’s exact test. P<0.05 was considered to indicate a statistically significant result. All statistical analyses were performed using SPSS 18.0 software (SPSS, Inc., Chicago, IL, USA).

## Results

### Morphology and roughness

**Figure 1.**
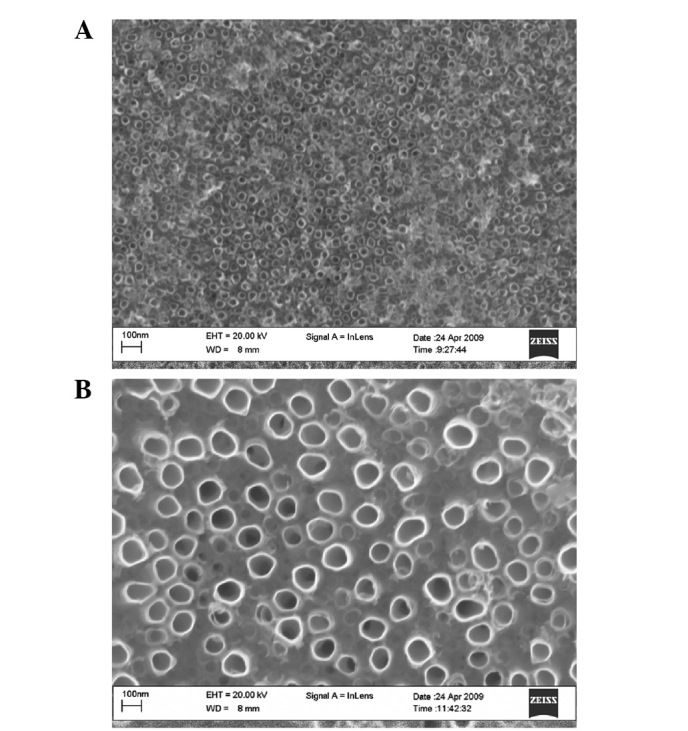
Morphological surface of Ti2448 alloy following anodization. (A) 30-nm and (B) 90-m titanium oxide films. Samples with a 30-nm nanoporous film exhibited a greater number of overlapping microporous structures with microprojections compared to samples with a 90-nm nanoporous film.

Representative FESEM micrographs of the 30- and 90-nm nanoporous titanium oxide films on Ti2448 alloys are shown in Fig. 1. Samples with a 30-nm nanoporous film exhibited a greater number of overlapping microporous structures with microprojections than samples with a 90-nm nanoporous film. As shown in Table II, the anodized titanium surfaces (30 and 90 nm) were rougher in comparison with the unanodized control titanium surface (P<0.05). However, no significant difference was observed in surface roughness between the 30- and 90-nm nanoporous films (P<0.05).

### Cell adhesion and morphology

**Figure 2.**
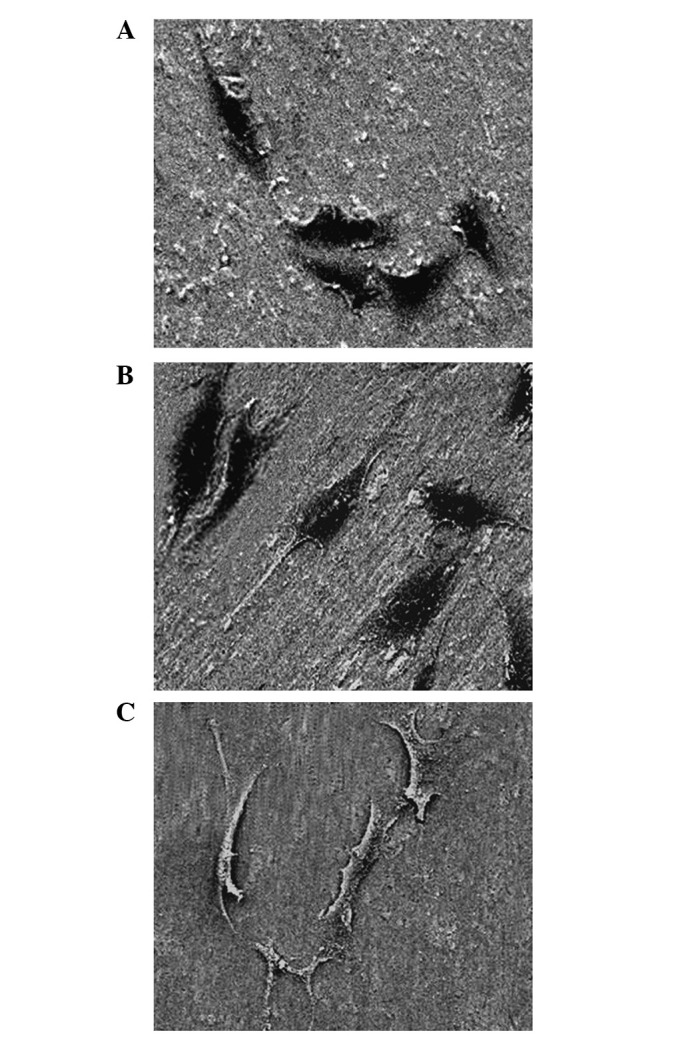
Low-magnification FESEM images of adhered osteoblasts on titanium surfaces following cell culture for 48 h. (A) Control unanodized titanium surface; (B) the 30-nm nanoporous surface; (C) the 90-nm nanoporous surface. FESEM, field emission scanning electron microscopy.

**Figure 3.**
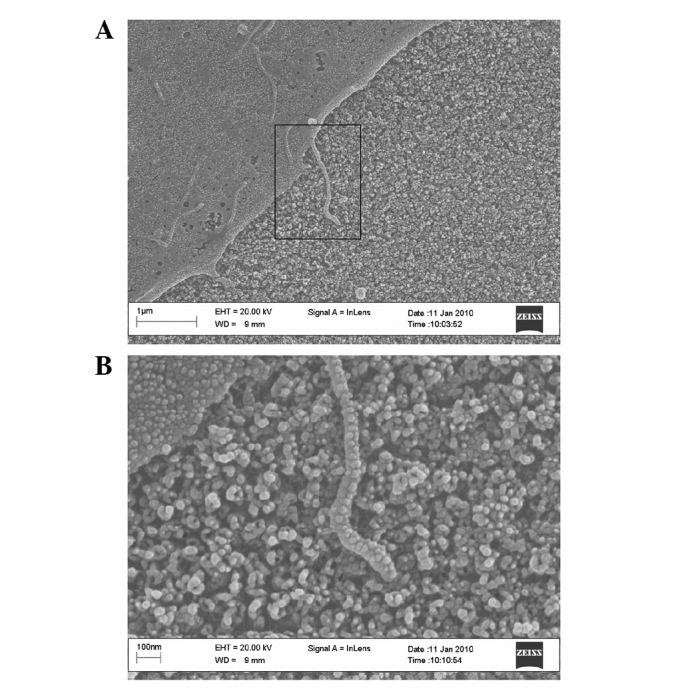
High-magnification FESEM images of adhered osteoblasts on the 30-nm nanoporous surface. The extracellular matrix (ECM) protein adhered densely to the top wall surface of the 30-nm nanoporous surface. FESEM, field emission scanning electron microscopy.

**Figure 4.**
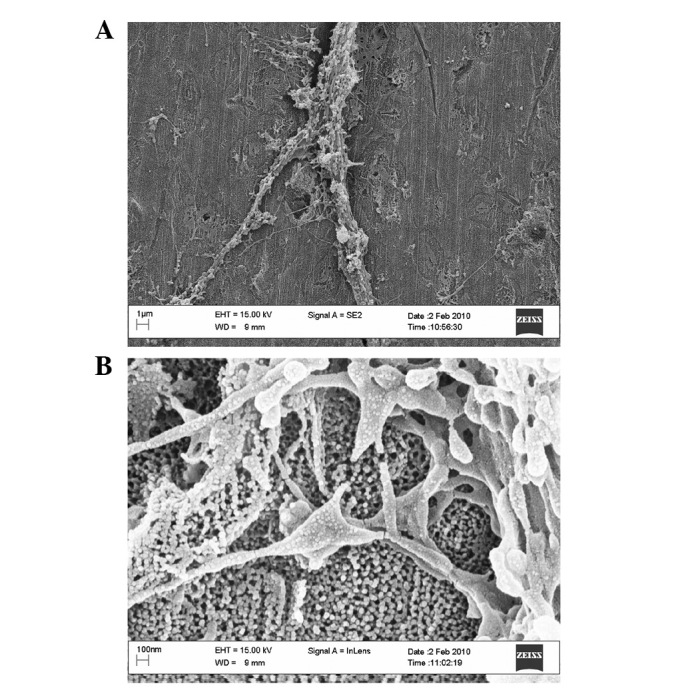
High-magnification FESEM images of adhered osteoblasts on 90-nm nanoporous surface. The extracellular matrix (ECM) protein adhered sparsely to the 90-nm nanoporous surface. FESEM, field emission scanning electron microscopy.

The morphology of the osteoblasts following cell culture in each group for 48 h is shown in Fig. 2. On the 30-nm nanoporous surface, the cells were densest on the overlapping structures and formed a polygonal shape without an evident wrapped edge. On the 90-nm nanoporous surface, cell adhesion was weaker than that observed on the unanodized control and 30-nm nanoporous surfaces, and a wrapped edge was evident. In addition, the cells exhibited a distorted and irregular shape. Under a high-magnification microscope, we observed that the extracellular matrix (ECM) protein adhered densely to the top wall surface of the 30-nm nanoporous surface (Fig. 3) and adhered sparsely to the 90-nm nanoporous surface (Fig. 4). We also observed the extension of filopodias from cells adhered to the nanoporous surface, which was mediated by ECM proteins, and that filopodias deviated around the nanoporous openings which were not covered by ECM proteins.

### MTT analysis

As shown in Table III, the MTT assay indicated that the viabilities of the cells on the 30-nm nanoporous surface were higher than those on the unanodized control and 90-nm nanoporous surfaces following 24 and 48 h of culture (P<0.05). The cell viabilities on the 90-nm nanoporous surface were lower than those on the control unanodized surface following 48 h of culture (P<0.05), while there was no significant difference between the cell viabilities of the 90-nm and control groups following 24 h of culture (P>0.05).

### Quantitative real-time RT-PCR analysis

**Figure 5.**
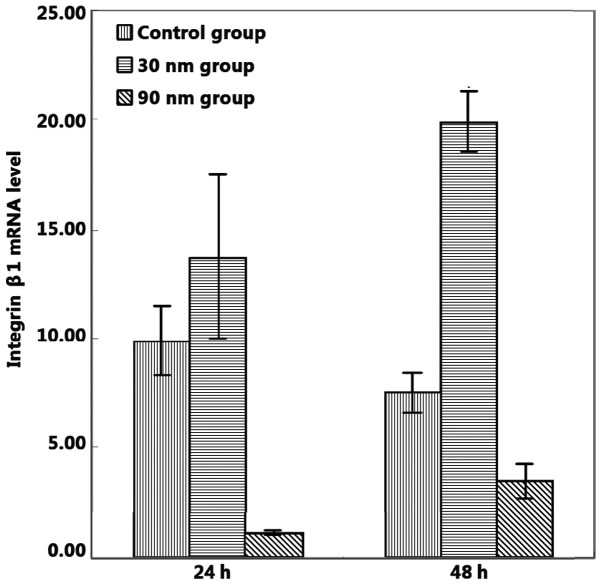
Integrin β1 mRNA expression levels following 24 and 48 h of cell culture.

**Figure 6.**
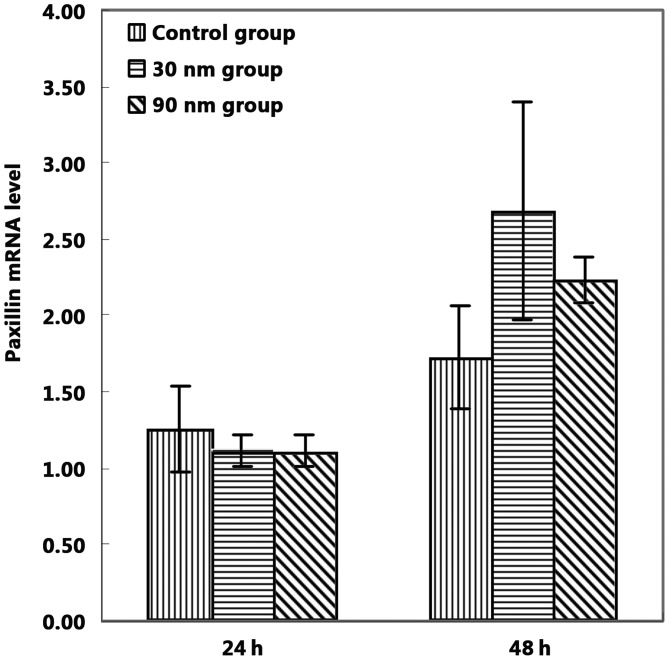
Paxillin mRNA expression levels following 24 and 48 h of cell culture.

Integrin β1 mRNA expression levels on the 30-nm nanoporous surface were significantly higher than those observed on the unanodized control and 90-nm nanoporous surfaces, following 48 h of cell culture (P<0.05; Fig. 5); however, no significant difference in the integrin β1 mRNA expression levels between the unanodized control and 30-nm nanoporous surfaces were observed following 24 h of cell culture (P>0.05). However, integrin β1 expression levels on the 90-nm nanoporous surface were evidently lower than those on the unanodized control surface following 24 and 48 h of cell culture (P<0.05). No statistically significant differences were observed in the expression levels of paxillin mRNA between the unanodized and anodized groups following 24 and 48 h of cell culture (Fig. 6).

## Discussion

In the last few decades, titanium metal and its alloys have been widely used as orthopedic and dental implants due to their good biocompatibility, mechanical properties and neutral interference with modern imaging techniques (26–28). To accelerate the initial rate of osseointegration, the topographic and chemical properties of implant surfaces should be considered and modified. The surface structure of the titanium implants is responsible for a good healing process. Therefore, numerous methods have been used to produce a rough implant surface, including titanium plasma spraying, blasting with aluminum oxide or other ceramic particulate materials, strong acid etching, and the potentiostatic or galvanostatic anodization of titanium (29). The majority of methods aim to form a micro/nanoporous structure, which may promote bone bonding or apposition of the implant surface (30).

In the present study, nanoporous titanium oxide films with two different pore sizes (30 and 90 nm) on Ti2448 alloy were formed by anodization in NH_4_F solution. The effects of the surface characteristics of different nanoporous titanium oxide films on the initial adhesion of osteoblasts was investigated. Following anodization, the surface morphology was examined using a Surftest Formtracer and FESEM. The roughness of the anodized titanium surfaces (30 and 90 nm) was significantly higher that of the unanodized titanium control surface. FESEM images demonstrated that the number of adhered cells on the 30-nm nanoporous surface was higher than those on the unanodized control and 90-nm nanoporous surfaces following 48 h of cell culture. In addition, cell adhesion on the 90-nm nanoporous surface was weaker than that on the unanodized control surface, indicating that the effects of surface micromorphology on initial cell adhesion may be more significant than the surface roughness. Our data provide strong evidence that the roughened surface of the 30-nm nanoporous film was more favorable for the initial adhesion of osteoblasts. Furthermore, we observed that the overlapped cells on the 30-nm nanoporous surface were the densest, suggesting that the surface demonstrated the characteristics of a typical bioactive surface with high surface energy. Results of the MTT assay indicated that cell viability on the 30-nm nanoporous surface following 24 and 48 h of cell culture was higher than those on the unanodized control and 90-nm nanoporous surfaces, which may have resulted from greater numbers of adhered osteoblasts and higher cell activities. This is in agreement with previous studies (7,9). *In vivo* studies have demonstrated an improved bone fixation on implants with a rough surface structure compared with implants with a smooth surface (27). A previous study has shown that integrin β1 is an important bridge for osteoblast adhesion on biomaterials (31). Zreiqat *et al* also demonstrated that higher expression levels of integrin β1 may contribute to the initial adhesion of osteoblastic cells to implant surfaces (31). Our results are in agreement with previously published studies with regard to the influence of integrin β1 on cell adhesion properties. Using real-time RT-PCR, we identified that integrin β1 mRNA expression levels on the 30-nm nanoporous surface following 24 h of cell culture were significantly higher than those on the control unanodized and 90-nm nanoporous surfaces. However, the 90-nm nanoporous surface demonstrated lower integrin β1 mRNA expression levels compared with the unanodized control surface following 24 and 48 h of cell culture. These results provide support for our hypothesis that nanoporous oxide films on Ti2448 alloy, particularly 30-nm nanoporous films, have a significant influence on osteoblast cell adhesion.

In conclusion, the results of the current study indicate that 30-nm nanoporous titanium oxide films on Ti2448 alloys may provide the optimum bioactive implant surface for the initial adhesion of osteoblasts. The 30-nm nanoporous film exhibited the characteristics of a typical bioactive surface. Thus, a Ti2448 alloy with a 30-nm nanoporous film is a promising implant-coating material for the promotion of bone formation, as well as for meeting the immediate implantation and early clinical load requirements. Further studies are required to determine whether the 30-nm nanoporous film on Ti2448 alloy has a strong effect on osteoblast cell proliferation, migration and differentiation.

## Figures and Tables

**Table I.** Real-time PCR primer sequences.

Gene	Access number (Gene Bank)	PCR primer sequences	Length (nucleotides)	Cycle number	PCR products (bp)
Integrin β1	X07979	5′-TTACGATGACGGTCTGGG-3′	18	46	122
		3′-AAATGGCTTGTGCTTGTT-5′	18		
Paxillin	P49023	5′-CTGCTGGCGGACTT-3′	14	23	120
		3′-TGGCACGGCAATCT-5′	14		
GAPDH	M32599	5′-GAGCCACATCGCTCAGACAC-3′	20	24	150
		3′-CATGTAGTTGAGGTCAATGG-5′	20		

**Table II.** Surface roughness for each group.

Group	Roughness		

	R_a_ (*μ*m)	R_z_ (*μ*m)	R_y_ (*μ*m)
Control group	0.28±0.11	1.37±0.26	1.88±0.17
30-nm group	0.76±0.03^a^	5.13±0.41^a^	6.14±0.29^a^
90-nm group	0.71±0.04^a^	4.97±0.51^a^	5.79±0.56^a^

R_a_, arithmetic mean deviation of the roughness profile; R_z_, mean peak-to-valley height; R_y_, maximum height of the roughness.

^a^ P<0.05 compared with the control group.

**Table III.** Optical density of each group at different time points examined using the MTT assay.

Group	Time points		

	12 h	24 h	48 h
Control group	0.015	0.062	0.093
30-nm group	0.017	0.133^a,b^	0.184^a,b^
90-nm group	0.014	0.053	0.068^a^

a P<0.05 compared with the control group;

b P<0.05 compared with the 90-nm group. MTT, 3-(4,5-dimethylthiazol-2-yl)-2,5-diphenyl tetrazolium bromide.
